# Blinded, bias‐controlled multi‐rater evaluation of human‐versus‐AI brain metastasis segmentation using a hybrid foundation‐model framework

**DOI:** 10.1002/mp.70538

**Published:** 2026-06-22

**Authors:** Yiding Han, Enze Zhu, Mhd Hasan AI Mekdash, Omar Awad, Piyush Pathak, Shixiao Liang, Daniel Allan Hamstra, Xizhe Zhang, Zaid Ali Siddiqui, Baozhou Sun

**Affiliations:** ^1^ Radiation Oncology Department Baylor College of Medicine Houston Texas USA; ^2^ Nanjing Medical University Nanjing Jiangsu China

**Keywords:** brain metastasis segmentation, foundation model, inter‐observer variability, preference‐based evaluation

## Abstract

**Background:**

Accurate segmentation of brain metastases (BM) is essential for diagnosis, stereotactic radiosurgery planning, and longitudinal assessment. However, manual contouring is time‐intensive, limiting clinical scalability, and exhibits substantial inter‐observer variability. This variability complicates objective assessment of automated segmentation methods and challenges interpretation of model performance.

**Purpose:**

To address these limitations, we developed TUM‐SAM, a hybrid foundation‐model framework for fully automated BM segmentation, and introduced a bias‐controlled, blinded multi‐rater evaluation paradigm to determine whether AI‐based BM segmentation has reached expert‐level performance and whether AI‐generated contours are preferred by human experts under unbiased assessment.

**Methods:**

TUM‐SAM integrates nnU‐Net‐based lesion detection with a tumor‐adapted Med‐SAM segmentation model to enable prompt‐free, fully automated segmentation. Training used 301 patients (2548 lesions), and external evaluation used an independent cohort of 105 patients (397 lesions). Segmentation accuracy was benchmarked against DeepMedic and nnU‐Net using Dice similarity coefficient (DSC) and 95th‐percentile Hausdorff distance (HD95). Two physicians contoured all external cases, and a third physician contoured a 20‐patient subset for a blinded, tumor‐level, multi‐rater preference study. Pairwise contour preferences were analyzed using a Bradley–Terry probabilistic model to obtain bias‐adjusted estimates of relative contour quality while accounting for rater‐specific tendencies and case difficulty.

**Results:**

In the external cohort, TUM‐SAM achieved a lesion‐wise detection sensitivity of 0.94 and outperformed DeepMedic and nnU‐Net across all tumor sizes, with a mean DSC of 0.84 and HD95 of 1.9 mm (nnU‐Net/DeepMedic: DSC < 0.70, HD95 > 3.3 mm). Across voxel‐wise evaluation, TUM‐SAM's geometric performance fell within the range of inter‐observer variability among physicians and was sensitive to reference construction. In contrast, in the blinded rater study, experts preferred TUM‐SAM–generated contours over individual physician contours in 81–87% of raw comparisons; Bradley–Terry analysis yielded conservative, bias‐corrected win probabilities of 55–56%, indicating consistent preference after adjustment for rater and case difficulty.

**Conclusion:**

Using a bias‐controlled, blinded multi‐rater evaluation framework, TUM‐SAM demonstrates brain metastasis segmentation quality that is consistently preferred by expert physicians, highlighting the limitations of agreement‐based voxel‐wise metrics under inter‐observer variability. These findings underscore the dependence of conventional evaluation on reference definition and support preference‐based assessment as a complementary approach for evaluating AI segmentation quality in BM MRI.

## 1 INTRODUCTION

Accurate segmentation of brain metastases (BM) is essential for diagnosis, longitudinal monitoring, and treatment planning. However, manual contouring of metastases remains labor‐intensive and highly variable, with substantial differences reported even among subspecialty‐trained radiologists and radiation oncologists. This variability is particularly pronounced for small, faintly enhancing, or irregular lesions.^1^, ^2^, ^3^, ^4^, ^5^

While deep‐learning segmentation models have demonstrated strong performance for many normal anatomical structures, automated tumor segmentation continues to be challenging.^6^, ^7^, ^8^, ^9^, ^10^ BM vary markedly in size, morphology, and contrast appearance, and a single MRI scan may contain dozens of discrete lesions. Traditional 3D convolutional neural networks (3D‐CNNs), including nnU‐Net and DeepMedic, require large, consistently annotated datasets and often generalize poorly across institutions, scanner types, or imaging protocols.^11^ These limitations hinder the development of reliable, broadly generalizable auto‐segmentation tools for clinical radiology.

Foundation models such as SAM and Med‐SAM have expanded medical image segmentation through large‐scale pretraining,^12^ but reliance on manual prompts and limited performance for small or heterogeneous metastases reduce clinical practicality.^13^ Auto‐prompting approaches have been proposed^14^, ^15^, ^16^ but have not consistently surpassed established 3D‐CNN baselines.

A further challenge within the BM segmentation literature is the evaluation paradigm itself. Most studies compare model‐generated contours to a single expert‐drawn “reference,” implicitly treating that contour as ground truth. This approach is problematic: substantial inter‐observer variability exists among experts,^17^ and disagreement with a single rater does not necessarily imply inferior segmentation quality.

Consensus algorithms such as the Simultaneous Truth and Performance Level Estimation (STAPLE) can partially reduce inter‐observer variability; however, they remain limited by the same underlying manual contouring biases, including slice‐wise editing constraints, time pressure, and ambiguity in delineating subtle enhancement.^18^ Importantly, neither single‐rater nor consensus‐based voxel‐wise metrics explicitly model expert preference or account for rater‐specific tendencies.

Consequently, voxel‐wise agreement metrics (Dice similarity coefficient, Hausdorff distance) even with consensus results fail to answer two key scientific and clinical questions:

1. Has AI‐based BM segmentation reached expert‐level performance within the range of inter‐observer variability?
2. If so, are AI‐generated segmentations preferred by experts under blinded, bias‐controlled evaluation?

To address these methodological and technical limitations, we introduce TUM‐SAM, a state‐of‐the‐art hybrid foundation‐model segmentation framework that combines nnU‐Net–based lesion detection with a tumor‐adapted, fine‐tuned Med‐SAM model to enable fully automated, prompt‐free segmentation.

In parallel, to overcome the fundamental limitations of single‐rater and consensus‐based evaluation, we propose a blinded multi‐rater evaluation framework analyzed using a Bradley–Terry probabilistic paired‐comparison model,^19^ representing a novel application of this methodology for assessing human‐versus‐AI segmentation quality in neuro‐oncology. The Bradley–Terry formulation efficiently aggregates pairwise comparisons and yields interpretable probabilities of preference while accounting for rater‐ and patient‐level variability.

Together, these contributions establish a clinically relevant and statistically principled approach for evaluating human‐versus‐AI segmentation quality and demonstrate that a hybrid foundation‐model framework can achieve high accuracy and strong expert preference in BM segmentation on MRI.

## 2 METHOD AND MATERIAL

### 2.1 Patient datasets

This study utilized two independent T1 post‐contrast MRI datasets: the UCSF dataset for model training and the TCIA GammaKnife‐Hippocampal dataset for external evaluation of brain metastasis detection and segmentation.

I. Training dataset—UCSF BMSR Dataset: The dataset for training was the University of California San Francisco Brain Metastasis Stereotactic Radiosurgery (UCSF BMSR) dataset,^20^ consisting of 306 patients with pre‐treatment MRI series. MRIs were acquired on a 1.5‐T GE SingaHDxt scanner using a post‐contrast 3D T1‐weighted SPGR (Spoiled Gradient Echo) sequence with TR  =  8.8 ms, TE  =  3.3 ms, flip angle  =  11°. All tumor contours in the UCSF dataset were manually delineated by expert clinicians as part of routine stereotactic radiosurgery planning.
II. Test dataset—The Cancer Imaging Archive (TCIA) Dataset: External evaluation was performed on the TCIA GammaKnife‐Hippocampal dataset,^21^ consists of 105 pre‐treatment imagings from patients undergoing Gamma Knife radiosurgery. MRIs were acquired with 3 D T1 weighted spoiled gradient echo (FLASH) sequence, post contrast, on a 1.5 T Siemens system: TR  =  11 ms, TE  =  3.2 ms, flip angle  =  20°. These cases were independently contoured by multiple physicians for this study, as described in Section 2.1.1, and were not used during model training.

#### 2.1.1 Physician segmentation for TCIA dataset

The external evaluation dataset consisted of 105 pre‐treatment MRI studies from the TCIA GammaKnife‐Hippocampal collection. Two independent physicians contoured the entire dataset:

1. Physician A: a junior attending radiation oncologist
2. Physician B: radiation oncologist with prior independent clinical practice, currently engaged in research

A third physician (Physician C, senior attending CNS radiation oncologist with >5 years as CNS (Brain) specialist) independently contoured a random subset of 20 patients to enable multi‐rater analysis. These three contour sets (A‐C) were also used to generate a STAPLE consensus segmentation for this subset.

### 2.2 Data preprocessing

All MRIs were converted from DICOM to NIfTI format, skull‐stripped,^23^ intensity‐normalized, and resampled to 1.0 × 1.0 × 1.5 mm. Very small detections (longest diameter in 3D lesion volume < 5 mm) were excluded due to unstable boundary definition, partial‐volume effects, and RANO non‐measurability.^22^ This study excludes 5% and 2% of the lesions as tumor cavities in each dataset, respectively. Connected‐component labeling was used to separate individual metastases; cases with no labeled lesions were excluded.

### 2.3 Prompt‐free TUM‐SAM

**FIGURE 1:**
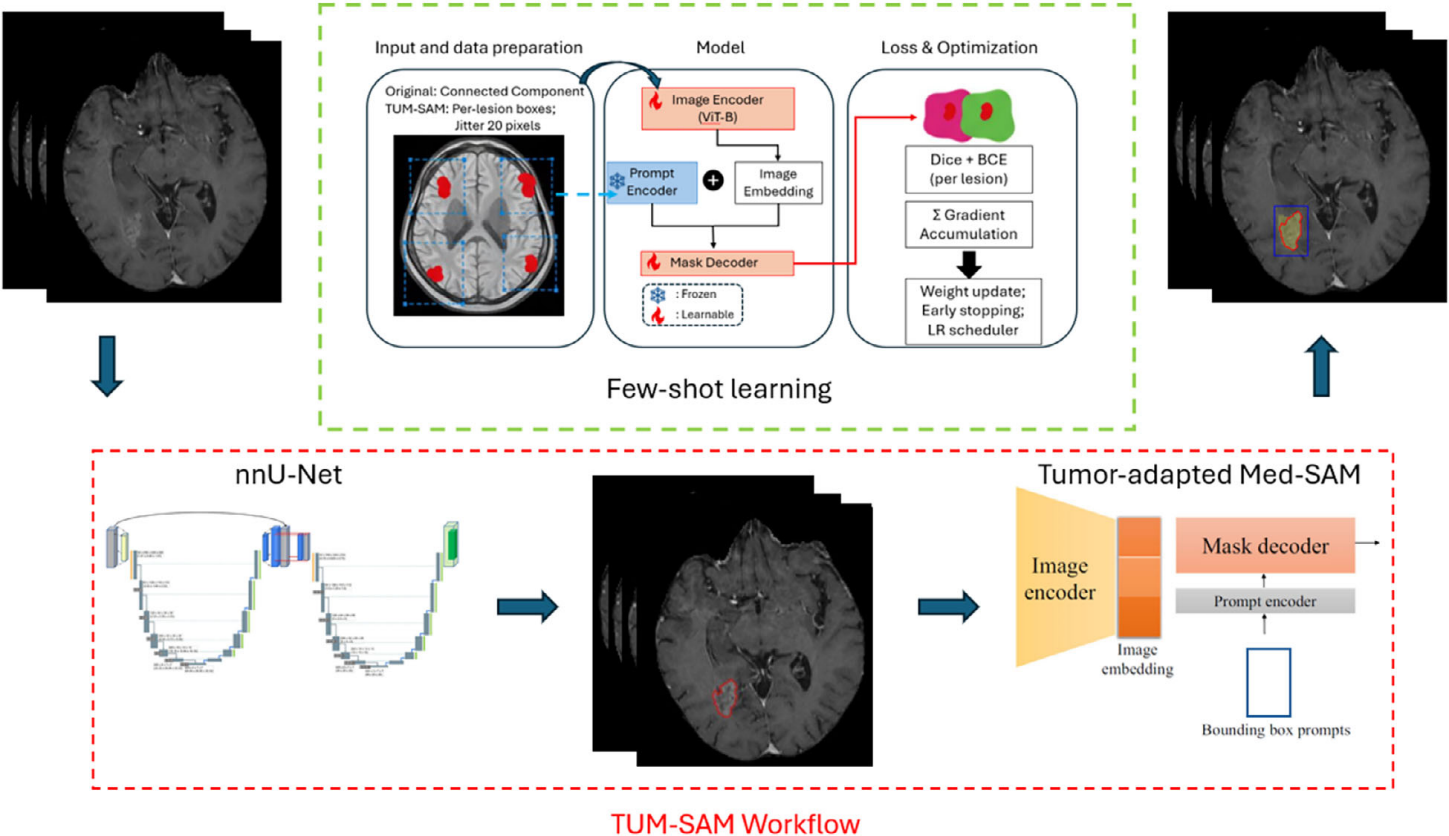
Overview of the proposed TUM‐SAM framework and tumor‐adapted Med‐SAM training strategy. Bottom (red dashed box): Inference workflow. nnU‐Net first performs fully automated brain metastasis detection on post‐contrast T1‐weighted MRI and generates coarse tumor masks. These detections are converted into per‐lesion bounding‐box prompts, which are passed to the tumor‐adapted Med‐SAM to refine each lesion and produce the final segmentation. Top (green dashed box): Tumor‐adapted Med‐SAM training architecture. Each metastasis is processed independently using its own bounding‐box prompt, enabling multi‐instance learning within a single image. The Image Encoder (ViT‐B) and Mask Decoder are fine‐tuned, while the Prompt Encoder remains frozen. Per‐lesion DSC and binary cross‐entropy (BCE) losses are aggregated via gradient accumulation to support efficient few‐shot training.

Figure 1 illustrates the proposed TUM‐SAM framework. The image series is first processed by the nnU‐Net model,^1^ which identifies tumor candidates and generates an initial segmentation. For each slice containing a detected tumor, a rectangular bounding‐box prompt was automatically generated by adding a 15‐pixel margin to the minimum and maximum x‐ and y‐axis boundaries of the tumor region. These prompt‐augmented images are then passed through the tumor‐adapted Med‐SAM model, which refines the segmentation and produces the final tumor contours.

The tumor‐adapted Med‐SAM model, derived from Med‐SAM,^14^ consists of three primary components: an Image Encoder (ViT‐Base), a Prompt Encoder, and a Mask Decoder. The Image Encoder processes each resized MRI slice to generate a compact image representation. The Prompt Encoder transforms bounding‐box prompts into embeddings; this module was kept frozen during training to preserve its robust, object‐agnostic prompt‐handling capability. Finally, the Mask Decoder integrates the image and prompt embeddings to produce the final segmentation mask.

### 2.4 Tumor‐adapted Med‐SAM training

The TUM‐SAM training strategy fine‐tunes Med‐SAM^12^ for multi‐instance BM segmentation, addressing a key limitation of the original framework. Standard Med‐SAM training selects a single object per image and learns to segment only that region, which is suboptimal for BM where multiple discrete lesions frequently coexist on the same scan.

To fully leverage the multi‐lesion nature of the task, we adopted a training scheme in which every metastasis in an image is processed with its own bounding‐box prompt, and the corresponding losses are aggregated through gradient accumulation. This “per‐lesion” strategy enables the model to learn instance‐level segmentation for all tumors in a series while maintaining efficient batch‐wise optimization. During fine‐tuning, the Image Encoder and Mask Decoder were updated, whereas the Prompt Encoder remained frozen to preserve its generalizable prompt‐handling behavior.

Training used a combined DSC + Binary Cross‐Entropy (BCE) loss and the AdamW optimizer (learning rate 1×10^−^^4^, weight decay 0.01), with up to 150 epochs and early stopping. This configuration encourages accurate delineation of individual metastases while improving robustness across varying lesion sizes and appearances.

Training was performed on an AWS instance equipped with an NVIDIA L40S GPU. Fine‐tuning the tumor‐adapted Med‐SAM model required approximately 26 hours, and nnU‐Net training required an additional 24 hours, for a total TUM‐SAM training time of approximately 50 hours. Inference was completed in under 1 minute per patient.

To contextualize the clinical utility of TUM‐SAM, we benchmarked its performance against two established 3D convolutional architectures widely used in brain‐metastasis segmentation research: nnU‐Net and DeepMedic.^23^

### 2.5 Evaluation metrics

Because no absolute ground truth exists for brain metastasis delineation, all metrics were computed relative to pragmatic expert reference standards and should be interpreted as measures of agreement rather than true correctness.

Detection performance was assessed using lesion‐wise sensitivity and false‐positive rate per scan. Detection metrics were computed relative to two reference standards: (i) Physician A alone, consistent with prior single‐expert benchmarking, and (ii) the union of Physician A and Physician B annotations, to account for inter‐observer variability in lesion identification.

Segmentation performance was evaluated using Dice Similarity Coefficient (DSC) and 95th‐percentile Hausdorff Distance (HD95), computed relative to Physician A's contours. Physician B's segmentations were included to characterize inter‐physician variability and to contextualize whether AI performance falls within the range of normal expert disagreement.

Pairwise differences in voxel‐wise segmentation metrics were tested using the two‐sided Wilcoxon signed‐rank test. Confidence intervals for volume‐stratified performance curves were derived using patient‐level (cluster) bootstrap with bias‐corrected and accelerated (BCa) intervals and are shown for visualization; all statistical inference relied on the Wilcoxon framework.

### 2.6 Consensus reference generation with STAPLE

To construct a consensus reference standard, we applied STAPLE algorithm to the three physician segmentations. STAPLE treats each rater's mask as a noisy observation of an unknown latent “true” segmentation and jointly estimates:

(1) the voxel‐wise probability of the true contour, and
(2) each rater's operating characteristics (sensitivity and specificity).

Through iterative expectation–maximization, STAPLE integrates these parameters to produce a probabilistic consensus map representing the most likely true segmentation across the raters. The resulting probability map was thresholded at 0.5 to obtain a binarized consensus mask for downstream DSC and HD95 evaluation, consistent with the standard use of STAPLE as a probabilistic estimate of latent true segmentation.^24^ All performance comparisons—including those for individual physicians and the AI model—were evaluated against this STAPLE‐derived consensus reference.

### 2.7 Rater study design

Three physicians participated in the rater study, with each serving as an independent observer when evaluating contour sets that did not include their own segmentations. Using an in‐house blinded comparison platform, each rater performed tumor‐level pairwise comparisons of contour sets. Comparisons were restricted to metastases that were detected by all participating methods (AI and all physician raters). For example, when Physician C acted as the observer, comparisons included AI vs. Physician A, AI vs. Physician B, and Physician A vs. Physician B. In each trial, the rater was required to select the superior contour for the entire 3D lesion volume; ties were not permitted. Contour source identity was fully masked, such that the observer was not informed whether a contour originated from TUM‐SAM or from a specific physician. In addition, left‐right display order was randomized to minimize presentation bias. The resulting binary preference data were analyzed using a Bradley–Terry paired‐comparison framework to estimate a latent contour‐quality score for each method while accounting for patient‐level variability.

#### 2.7.1 Model formulation

Each method $i\in \{AI,P_{1},P_{2},P_{3}\}$ was assigned a latent ability parameter $\beta_{i}$.

The Bradley–Terry model^25^, ^26^ specifies the probability that method i is preferred over method j:

(1) $$P\left(i\;\mathrm{beats}\;j\right)=\frac{e^{\beta_{i}}}{e^{\beta_{i}}+e^{\beta_{j}}}=\frac{1}{1+e^{-\left(\beta_{i}-\beta_{j}\right)}}$$

Thus, preference depends solely on the difference $\beta_{i}-\beta_{j}$.

A logistic‐regression design matrix encoded each comparison using +1 for the chosen (winning) method, –1 for the unchosen (losing) method, and 0 for all others. To mitigate systematic biases, we incorporated patient‐level and rater‐level dummy variables as additional covariates.

Because the dataset contains near‐perfect wins for some pairs (causing “complete separation”), we used a ridge‐regularized logistic regression:

(2) $$\hat{\beta }=\arg\max_{\beta }\left[\sum_{k}\log P\left(y_{k}|\beta \right)-\frac{\lambda }{2}\sum_{i}\beta_{i}^{2}\right]$$

where λ is the ridge penalty.

For presentation, the estimated $\beta_{i}$ values were mean‐centered to avoid arbitrary dependence on a reference method and to facilitate direct comparison across contouring approaches. To quantify uncertainty, we performed cluster bootstrap resampling at the patient level (200 iterations). For each bootstrap replicate, the full Bradley–Terry model was re‐fit and the ability parameters β were re‐estimated.

## 3 RESULTS

**FIGURE 2:**
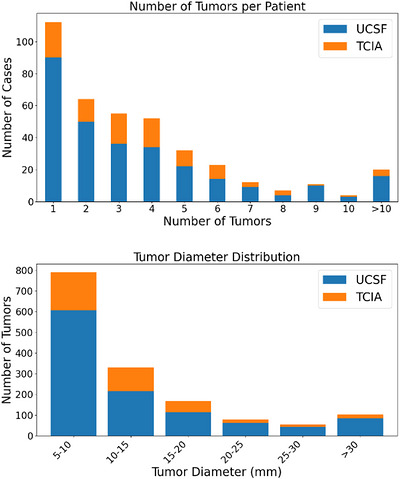
The metastasis statistics for the datasets used in this study. Tumor diameter was defined as the maximum diameter of the 3D lesion volume.

The characteristics of the datasets used in this study, after application of the exclusion criteria described in Section 2.2, are summarized in Figure 2 and Table 1. Across both datasets, the median number of tumors per patient was three, with modest variation between institutions. Tumor‐count distributions differed significantly between UCSF and TCIA (Kruskal–Wallis H = 5.442, *p* = 0.02), reflecting expected differences in clinical case mix and metastatic burden. In contrast, tumor‐size distributions were similar across datasets (Kruskal–Wallis H = 2.562, *p* = 0.10), indicating comparable lesion‐size profiles. The 20‐patient subset used for the multi‐rater study was randomly selected from the TCIA cohort after application of the eligibility criteria. This subset included 14 female and 6 male patients, with a mean age of 60 ± 11 years, a median of 4 tumors per patient (IQR = 3), and a median tumor volume of 0.40 cc (IQR = 0.816).

**TABLE 1:** Patient characteristics across datasets.

Dataset	# Patients	Female	Male	Age	Median tumors	Tumor volume(CC)
UCSF	306	178	128	61 ± 12	3 (IQR = 4)	0.234 (IQR = 0.723)
TCIA	104	70	34	63 ± 10	3 (IQR = 3)	0.363 (IQR = 0.895)

### 3.1 Tumor detection accuracy

Table 2 summarizes the tumor‐detection performance of TUM‐SAM on the external 105 patients cohort under two reference standards: (1) the union of Physician A and Physician B segmentations (upper rows), and (2) Physician A alone as a single‐expert reference (lower rows).

**TABLE 2:** Detection performance of TUM‐SAM compared to Union (PhysicianA ∪ Physician B) and single expert physician A including lesion‐wise sensitivity and false positives per scan with 95% CI.

Reference: Physician A ∪ Physician B	Lesion‐wise sensitivity (95% CI)	FP per Scan (95% CI)
TUM‐SAM	85.6% (85%‐86%)	0.35 (0.2‐0.5)
Deep‐Medic	72% (71%‐73%)	1.15 (0.87‐1.7)
Physician A	88.5% (88%‐89%)	None
Physician B	79.8% (79.4%‐80.2%)	None
Reference: Physician A	Lesion‐wise sensitivity (95% CI)	FP per Scan (95% CI)
TUM‐SAM	94% (93.5%‐94.5%)	0.89 (0.84‐0.93)
Deep‐Medic	80% (79%‐81%)	1.52 (1.21‐1.84)
Physician B	86.7% (86.2%‐87.2%)	0.84 (0.6‐1.1)

Physician A was selected as the single‐expert reference to enable direct comparison with prior studies and to contextualize TUM‐SAM's performance relative to an experienced attending radiation oncologist.

Using the union reference, TUM‐SAM achieved a lesion‐wise sensitivity of 85.6%, with a low mean false‐positives (FP) of 0.35 per scan. When evaluated against Physician A alone, TUM‐SAM demonstrated substantially higher sensitivity, achieving a lesion‐wise sensitivity of 94.0% with 0.89 false positives per scan. This discrepancy primarily reflects inter‐observer variability in the identification of small lesions, particularly when distinguishing subtle metastases from vascular structures or background enhancement.

### 3.2 Tumor segmentation accuracy

**FIGURE 3:**
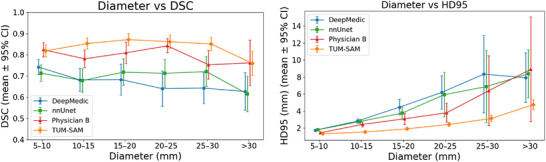
*Geometric segmentation accuracy of TUM‐SAM (orange), DeepMedic (blue), nnU‐Net (green), and Physician B (red) across lesion diameter categories, evaluated using Physician A as the reference standard*. Left: DSC similarity coefficient (mean ± 95% BCa CI). Right: 95th‐percentile Hausdorff distance, HD95 (mean ± 95% BCa CI). Vertical bars denote BCa bootstrap confidence intervals. Smaller HD95 and higher DSC indicate better geometric agreement.

Figure 3 illustrates the geometric segmentation performance of TUM‐SAM (orange), Physician B (red), DeepMedic (blue), and nnU‐Net (green) across tumors of varying maximum diameters, using Physician A as the reference standard. Across all diameter ranges, TUM‐SAM consistently achieved high DSC scores and low HD95 values, indicating strong geometric agreement with the clinical reference.

Table 3 summarizes the overall performance averaged across all lesions. TUM‐SAM demonstrated significantly better geometric accuracy than both 3D‐CNN–based models and the individual physician, achieving the highest DSC (0.84 [95% CI: 0.82‐0.85]) and the lowest HD95 (1.9 mm [95% CI: 1.7‐2.0]). The performance of nnU‐Net and DeepMedic was consistent with previously reported results from cross‐dataset generalization studies, with reported DSCs of 0.675 ± 0.168 for nnU‐Net^27^ and 0.69 ± 0.2 for DeepMedic.^28^

**TABLE 3:** Summary of overall averaged geometric segmentation performance of all lesions for TUM‐SAM, nnU‐Net, DeepMedic, and Physician B relative to Physician A. TUM‐SAM achieves the highest DSC and lowest HD95, outperforming both deep‐learning baselines and the independent physician.

	TUM‐SAM [95% CI]	nnU‐net [95% CI]	Deep‐Medic [95% CI]	PhysicianB [95% CI]
Dice	0.84 [0.82‐0.85]	0.69 [0.67‐0.71]	0.68 [0.66‐0.70]	0.8 [0.79‐0.82]
HD95 (mm)	1.9 [1.7‐2.0]	3.34 [2.9‐3.8]	3.54 [3.1‐4.0]	2.5 [2.2‐2.8]

### 3.3 TUM‐SAM vs. multi‐physician consensus map

Table 4 summarizes lesion‐wise detection and geometric segmentation performance for TUM‐SAM and three physicians, evaluated relative to a STAPLE‐derived consensus map constructed from the three physician contours for the 20‐patient subset.

**TABLE 4:** Summary of overall detection and geometric segmentation performance for TUM‐SAM and three physicians relative to consensus map from physicians’ contouring.

	Lesion‐wise sensitivity [95% CI]	FP per Scan	Dice [95% CI]	HD95 (mm) [95% CI]
TUM‐SAM	92% [90‐94%]	0.89	0.84 [0.82‐0.86]	2.0 [1.6‐2.4]
PhycisianA	100% [98‐100%]	0.05	0.95 [0.94‐0.97]	1.3 [0.9‐1.6]
PhycisianB	90% [88‐93%]	0.68	0.79 [0.76‐0.82]	2.5 [2.0‐2.9]
PhycisianC	99% [97‐100%]	0	0.97 [0.95‐0.98]	1.1 [0.68‐1.5]

When evaluated against the consensus reference, TUM‐SAM achieved a lesion‐wise sensitivity of 92% (95% CI: 90–94%) with 0.89 false positives per scan, a mean DSC of 0.84 (95% CI: 0.82–0.86), and an HD95 of 2.0 mm (95% CI: 1.6–2.4). Relative to the STAPLE reference, TUM‐SAM showed detection performance comparable to Physician B, while outperforming Physician B on segmentation metrics, with higher DSC and lower HD95.

Physician A and Physician C exhibited higher DSC values and lower HD95 relative to the STAPLE‐derived consensus than Physician B and TUMSAM. This does not imply superior absolute contour quality; rather, it indicates that their segmentations were more similar to the consensus estimated from the joint agreement structure of the three raters. Accordingly, performance differences relative to the STAPLE reference should be interpreted as differences in agreement with the consensus construction, not as direct evidence of ground truth accuracy.

### 3.4 Rater study

In raw pairwise comparisons, TUM‐SAM was favored over Physician A in 86.8% of trials, over Physician B in 81.2%, and over Physician C in 85.7%.

**FIGURE 4:**
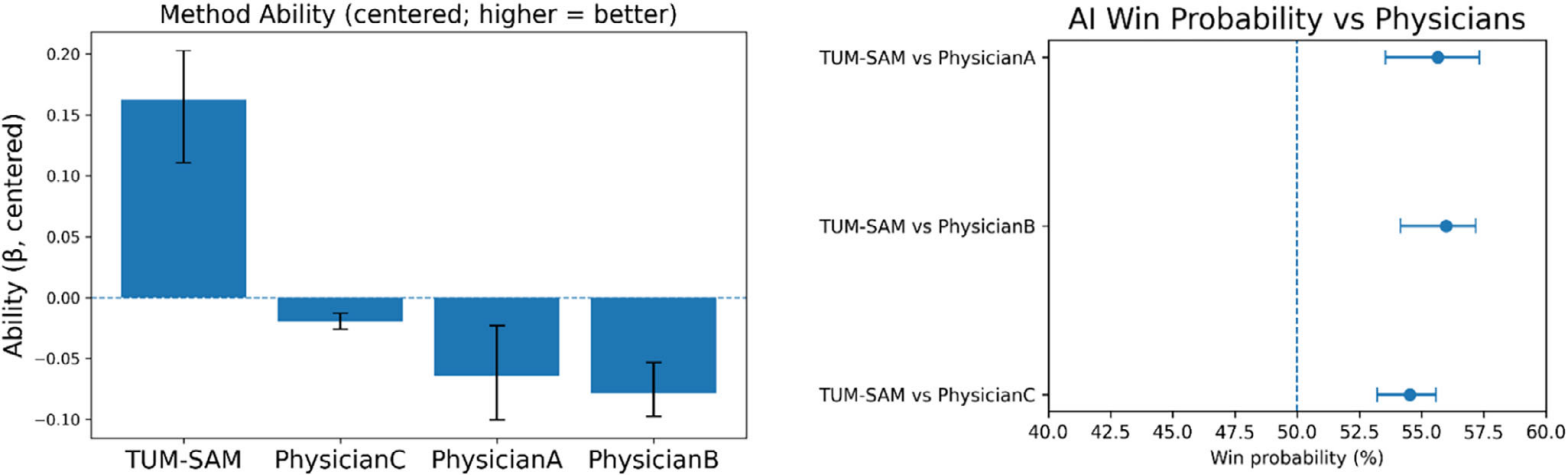
Physician rater–study results comparing contour quality between TUM‐SAM and three independent radiation oncologists. (Left) Bradley–Terry ability scores (centered, mean = 0) summarize the overall likelihood of producing the preferred contour across all pairwise comparisons (mean ± 95% BCa CI). Higher scores indicate superior perceived contour quality. (Right) Pairwise win‐probabilities show the estimated probability (mean ± 95% BCa CI) that TUM‐SAM's contour would be preferred over each physician's contour in a head‐to‐head comparison.

The Bradley–Terry model (Equation 2; Figure 4) demonstrated that TUM‐SAM achieved the highest overall ability score (Higher scores indicate superior perceived contour quality), outperforming all three physicians. Its centered ability estimate was positive and substantially higher than all physicians.

Consistent with this finding, pairwise win‐probability estimates derived from Equation 1 favored the TUM‐SAM. TUM‐SAM's contours were preferred over those of Physician A in 55.6% (95% CI: 53.5%–57.3%), over Physician B in 56.0% (95% CI: 54.2%–57.2%), and over Physician C in 54.5% (95% CI: 53.2%–55.6%) of comparisons.

## 4 DISCUSSIONS

The primary contribution of this study is the introduction a novel application of the blinded, preference‐based evaluation framework for human‐versus‐AI segmentation using a Bradley–Terry paired‐comparison model. Rather than relying on voxel‐wise agreement with a single reference contour, this approach directly measures expert preference, accounts for rater‐specific bias and case difficulty, and yields an interpretable latent estimate of contour quality.

Using this framework, we demonstrate that TUM‐SAM–generated brain metastasis segmentations are consistently preferred by expert physicians, even after conservative model‐based adjustment. Complementing this evaluation paradigm, TUM‐SAM provides a clinically scalable, prompt‐free hybrid foundation‐model solution that achieves voxel‐wise performance within the range of inter‐observer variability, enabling a meaningful and bias‐controlled human‐versus‐AI comparison.

Despite outperforming prior atlas‐based approaches,^29^ TUM‐SAM's sensitivity/specificity remained inferior to the performance of the attending radiation oncologist, as measured against the three‐physician consensus on the 20‐patient subset (sensitivity 99–100%, FP/scan 0.05, Table 4). This gap is primarily attributable to very small lesions, for which AI systems continue to struggle to distinguish faint enhancement from vascular structures and background noise—a well‐recognized challenge in automated BM detection.^30^, ^31^, ^32^, ^33^, ^34^ Notably, according to Tables 2 and 4, TUM‐SAM outperformed the rater with more limited recent clinical contouring exposure, underscoring the importance of sustained clinical experience for achieving expert‐level detection accuracy.

For segmentation, TUM‐SAM demonstrated significantly higher accuracy than traditional 3D CNN models, including DeepMedic and nnU‐Net, across all tumor‐size ranges (Figure 3). When compared with Physician B, TUM‐SAM's voxel‐wise performance fell within the expected range of inter‐observer variability (Figure 3, Table 3), highlighting that conventional overlap metrics alone cannot fully characterize differences in contour quality between human readers and AI. This motivated the use of a blinded rater study to obtain a bias‐corrected, preference‐based assessment of TUM‐SAM relative to expert physicians.

To further contextualize voxel‐wise evaluation under reduced reference uncertainty, segmentation performance was also assessed relative to a multi‐physician consensus map. As shown in Table 4, Physician A and Physician C achieved similarly higher DSC scores and lower HD95 values against the STAPLE‐derived consensus than Physician B and TUM‐SAM. Under this evaluation paradigm, TUM‐SAM demonstrated geometric performance comparable to the inter‐observer range but did not exceed the consensus‐aligned physicians. Notably, however, despite this apparent advantage for physician contours in consensus‐based voxel‐wise metrics, the blinded expert rater study (Figure 4) consistently preferred TUM‐SAM‐generated segmentations over individual physician contours in direct pairwise comparisons. This divergence reflects an inherent limitation of consensus‐based evaluation: when two physicians agree more closely with each other, the consensus map is pulled toward their shared delineation pattern and correspondingly penalizes the more discordant contour. Consequently, consensus‐based voxel‐wise evaluation predominantly measures conformity to prevailing physician behaviors—including slice‐wise contouring workflows, interpolation algorithms embedded in treatment planning systems, and institution‐specific contouring conventions—rather than independent contour quality.

Although the raw preference rates favored TUM‐SAM strongly (81–87% win rates against individual physicians), the Bradley–Terry model produced more conservative win probabilities of approximately 55–56% (Figure 4). This discrepancy is expected because the two summaries measure fundamentally different quantities. Raw win rates represent the unadjusted proportion of comparisons won by the AI, whereas the Bradley–Terry model enforces a single transitive ranking across all four methods (AI and three physicians) and adjusts simultaneously for rater‐specific biases and patient‐level difficulty. As a result, raw opponent‐specific comparisons may imply a different relative ordering among physicians than the global ranking inferred by the model. In our dataset, the physicians exhibited asymmetric performance relative to one another, requiring the model to shrink pairwise probabilities toward a coherent global solution. Thus, the Bradley–Terry probabilities should be interpreted as ability‐adjusted estimates of head‐to‐head performance, whereas the raw percentages illustrate the magnitude of observed preference. Together, they demonstrate that TUM‐SAM is consistently preferred over each physician, even after conservative model‐based adjustment.

**FIGURE 5:**
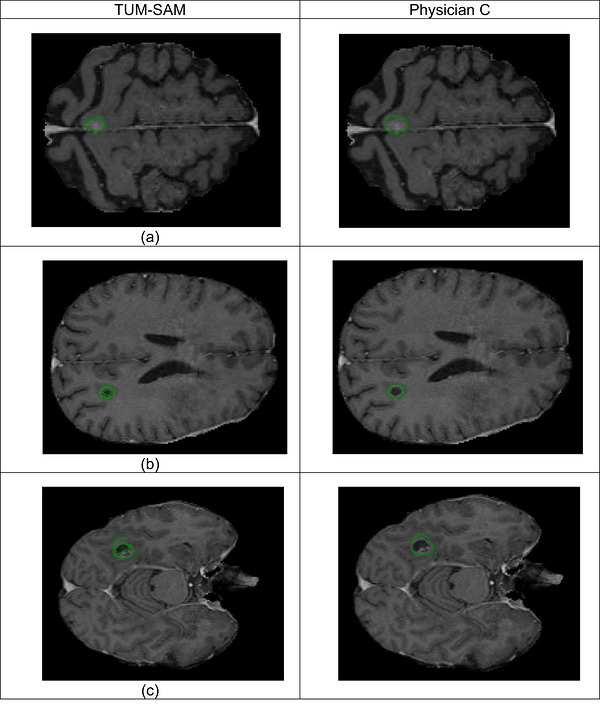
*Illustrative examples from the rater study*. Figure 5 (a): top row shows a case in which both raters (Physician A and Physician B) preferred the TUM‐SAM generated contour over that of Physician C. Figure 5 (b) and (c): the second and third rows show cases in which all raters judged the TUM‐SAM contour to be inferior to Physician C's contour when TUM‐SAM only contour only the hyperintense enhancing rim.

Figure 5 presents representative examples in which raters judged TUM‐SAM to either outperform or underperform relative to human experts. When TUM‐SAM was preferred as shown in Figure 5 (a) (top row) in over 80% cases, the advantage likely reflected its ability to optimize boundary placement from local voxel‐wise intensity changes throughout the lesion margin. By contrast, physician contouring in routine clinical practice is not performed as exhaustive voxel‐by‐voxel boundary optimization, because that level of manual refinement is impractical. Conversely, expert contours were occasionally favored over the model, shown in Figure 5 (b) and (c) (middle and bottom rows), particularly for metastases containing internal low‐signal regions—such as centrally necrotic, cystic, or hemorrhagic lesions. In these cases, TUM‐SAM sometimes segmented only the hyperintense enhancing rim, yielding a characteristic “donut‐like” contour that underestimated the hypo‐intense core. Although such cases could be mitigated with additional post‐processing or shape priors, their consistent appearance suggests that centrally necrotic metastases occupy a distinct region of the model's embedding space, raising the possibility that these representations may be leveraged for downstream tasks such as tumor subtyping or treatment‐response prediction. It also raises caution in deploying even “superhuman” AIs without careful evaluation, given the potential for systemic biases.

An important limitation of this study is the scalability of the blinded multi‐rater evaluation. Although the Bradley–Terry framework provides a rigorous, bias‐corrected assessment of human‐versus‐AI contour quality, obtaining the underlying multi‐readers comparisons is labor‐intensive: in our study, contour evaluation for 20 patients required approximately one hour of review time per physician. Scaling this paradigm to the hundreds or thousands of cases typically required for regulatory evaluation—such as FDA submissions—would be logistically and financially challenging.

## 5 CONCLUSION

In conclusion, the TUM‐SAM framework achieved expert‐level detection and segmentation performance for brain metastases by bridging 3D‐CNN–based lesion detection with foundation‐model–based refinement. For lesion detection, the model achieved 94% sensitivity while maintaining a low false‐positive burden. Across conventional voxel‐wise metrics, TUM‐SAM demonstrated strong geometric performance relative to established deep‐learning baselines and within the range of inter‐observer variability under both single‐rater and consensus‐based reference constructions. Importantly, TUM‐SAM–generated segmentations were consistently preferred by experts in blinded pairwise comparisons, with Bradley–Terry modeling demonstrating a significantly higher latent quality score than all human raters. Together, these findings indicate that TUM‐SAM provides a robust, physician‐in‐the‐loop solution for brain metastasis segmentation, while also highlighting the limitations of agreement‐based evaluation and the value of preference‐based assessment under inter‐observer uncertainty.

## CONFLICT OF INTEREST STATEMENT

The authors declare no conflicts of interest.

## Data Availability

Research data are stored in an institutional repository and will be shared upon request to the corresponding author.
